# OVOL2 antagonizes TGF-β signaling to regulate epithelial to mesenchymal transition during mammary tumor metastasis

**DOI:** 10.18632/oncotarget.17031

**Published:** 2017-04-11

**Authors:** Rong-Si Wu, Jing-Jing Hong, Jia-Fa Wu, Shen Yan, Di Wu, Na Liu, Qing-Feng Liu, Qiu-Wan Wu, Yuan-Yuan Xie, Yun-Jia Liu, Zhong-Zheng Zheng, Err-Cheng Chan, Zhi-Ming Zhang, Bo-An Li

**Affiliations:** ^1^ State Key Laboratory of Cellular Stress Biology, Innovation Center for Cell Signaling Network, School of Life Sciences, Xiamen University, Xiamen, Fujian, China; ^2^ The First Affiliated Hospital, Xiamen University, Xiamen, Fujian, China; ^3^ Engineering Research Center of Molecular Diagnostics, Ministry of Education, School of Life Sciences, Xiamen University, Xiamen, Fujian, China; ^4^ College of Food and Bioengineering, Henan University of Science and Technology, Luoyang, China; ^5^ Department of Medical Biotechnology and Laboratory Science, Chang Gung University, Taoyuan, Taiwan

**Keywords:** mammary tumor, EMT, OVOL2, TGF-β signaling, Smad4

## Abstract

Great progress has been achieved in the study of the role of TGF-β signaling in triggering epithelial-mesenchymal transition (EMT) in a variety of cancers; however, the regulation of TGF-β signaling during EMT in mammary tumor metastasis has not been completely defined. In the present study, we demonstrated that OVOL2, a zinc finger transcription factor, inhibits TGF-β signaling-induced EMT in mouse and human mammary tumor cells, as well as in mouse tumor models. Data from the Oncomine databases indicated a strong negative relationship between OVOL2 expression and breast cancer progression. Moreover, our experiments revealed that OVOL2 inhibits TGF-β signaling at multiple levels, including inhibiting Smad4 mRNA expression and inducing Smad7 mRNA expression, blocking the binding between Smad4 and target DNA, and interfering with complex formation between Smad4 and Smad2/3. These findings reveal a novel mechanism that controls the TGF-β signaling output level *in vitro* and *in vivo*. The modulation of these molecular processes may represent a strategy for inhibiting breast cancer invasion by restoring OVOL2 expression.

## INTRODUCTION

Tumor invasion and metastasis are the result of a complex process that involves local invasion, intravasation, transport, extravasation, micro-metastasis formation, and colonization [1, 2]. The transformation of cells to a fibroblastic phenotype is pivotal for local invasion and is the first step required for cancer cells to successfully metastasize. Accumulating studies suggest that the induction of the epithelial to mesenchymal transition (EMT) plays a key role in cancer cell transformation and progression [3, 4]. EMT is a process that is associated with marked changes in cell adhesion, polarity and migratory properties and is typically characterized by the downregulation of epithelial markers, such as E-cadherin, and the upregulation of mesenchymal markers, such as Vimentin [3, 5–9]. In contrast, the mesenchymal to epithelial transition (MET) may promote the growth of metastatic cancer cells at secondary sites [10]. EMT and MET are very important in tumor invasion and metastasis, as well as in tissue development and remodeling processes, such as secondary palate formation, mesoderm and neural crest formation, heart valve development, and wound healing [3, 5–9, 11]. Due to the complex and dynamic nature of EMT and MET, multiple signaling pathways that are important for both normal development and cancer development, including TGF-β, Wnt/β-catenin, Notch, EGF, HGF, FGF, and HIF, have been implicated in the regulation of these processes [6, 8, 12]. These signaling pathways directly or indirectly activate many EMT-related transcription factors, such as SNAIL (SNAIL1), SLUG (SNAIL2), TWIST1/2, EF1/ZEB1, SIP1/ZEB2, and E47, which subsequently inhibit E-cadherin production [6].

TGF-β signaling plays a pivotal role in the development of normal tissues and cancers through the control of proliferation, differentiation, apoptosis, adhesion, invasion, and the cellular microenvironment [13–16]. TGF-β signaling is transduced by a heteromeric complex of TβRI and TβRII, cell-surface serine–threonine kinase receptors, and the intracellular signal transducers Smad2 and Smad3. In response to TGF-β ligands, TβRII transphosphorylates TβRI, which subsequently mediates the phosphorylation of the receptor-regulated Smad2 and Smad3 (R-Smads). Phosphorylated Smad2/3 associates with the common partner Smad4 (Co-Smad) and translocates to the nucleus to regulate gene expression [17, 18]. Smad7 is an inhibitory Smad (I-Smad) that inhibits TGF-β signaling through multiple mechanisms. Importantly, Smad7 binds to activated type I receptors and competes with R-Smads for receptor binding, resulting in the repression of TGF-β signaling [19, 20]. The biological function of TGF-β in epithelial cells is complicated. TGF-β potently inhibits the proliferation of epithelial cells [21]. Transgenic overexpression of active TGF-β1 in the mouse mammary epithelium results in hypoplastic mammary glands, which fail to undergo oncogene- or carcinogen-induced mammary carcinogenesis [22–24]. Similarly, the overexpression of TGF-β1 in keratinocytes of a chemically induced mouse skin tumor model suppresses the formation of skin tumors. However, once tumor formation is completed, TGF-β1 enhances tumor progression to a highly invasive mesenchymal cell phenotype [25]. For example, the introduction of dominant-negative TGF-β type II receptors (TβRII) into Ha-Ras–induced mammary tumor cells suppresses the formation of metastases by primary tumors by preventing EMT [26]. Thus, TGF-β signaling has both tumor-suppressive and tumor-promoting functions [27].

The *Ovo* gene family encodes evolutionarily conserved zinc-finger transcription factors. Three Ovo homologues are present in mammals, which are designated Ovol1, Ovol2 and Ovol3 in mice and OVOL1, OVOL2 and OVOL3 in humans. Our laboratory and other groups have cloned the mammalian *Ovol2* gene [28, 29] and have identified its function in the development of the cranial neural tube, the heart, the placenta, keratinocytes and the mammary gland [28, 30–34]. Two recent studies demonstrate that OVOL2 inhibits EMT in the mouse mammary gland and human breast cancer cells through the transcriptional inhibition of ZEB1 [33, 35]. However, regulation of the EMT process by OVOL2 in breast cancer is not completely defined. Herein, we provide evidence to demonstrate that OVOL2 antagonizes TGF-β signaling at multiple levels of the signaling cascade, resulting in the inhibition of EMT during mammary tumor metastasis. Our study reveals a complex association between OVOL2 and the central EMT signaling pathway, which highlights the role of OVOL2 in the regulation of breast cancer malignant phenotypes.

## RESULTS

### OVOL2 inhibits TGF-β-induced EMT during mammary tumor metastasis and is a candidate metastasis suppressor in mammary tumors

**Figure 1:**
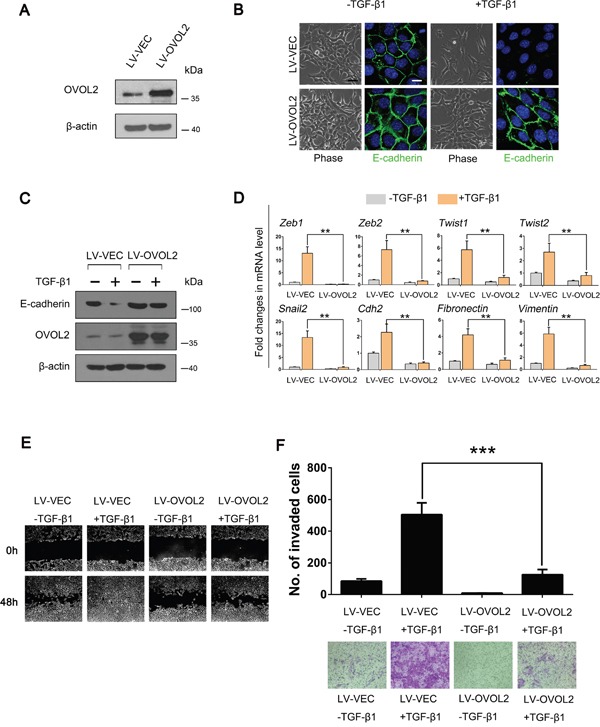
OVOL2 inhibits TGF-β-induced EMT during mammary tumor metastasis **(A)** Western blotting was used to detect OVOL2 protein levels in NMuMG cells treated with lentiviruses expressing a vector control (LV-VEC) and OVOL2 (LV-OVOL2). **(B)** Phase contrast and E-cadherin immunofluorescence images of control and OVOL2 overexpressing NMuMG cells undergoing TGF-β1 (5 ng/ml for 48 h)-induced EMT. Scale bars, 40μm (for bright field) and 10 μm (for E-cadherin). **(C)** Western blotting was used to detect the protein levels of OVOL2 and E-cadherin in control and OVOL2 overexpressing NMuMG cells. **(D)** Quantitative real-time PCR was used to measure the mRNA levels of EMT-related genes in control and OVOL2 overexpressing NMuMG cells with or without TGF-β1 (5 ng/ml 48 h) treatment. The real-time PCR values were normalized to the housekeeping gene *Gapdh*. The experiments were performed three times, each with real-time PCR performed in technical triplicate, and the data are presented as the mean ± SD. **P < 0.01, as indicated by Student's t-test. **(E)** The migration ability of the above cells was assessed using a wound-healing assay. The experiment was performed in triplicate. Representative images are shown. **(F)** The invasive ability of the above cells is presented as the total number of cells that entered the bottom invasion chamber, counted across eight fields. Each sample was measured in triplicate, and each experiment was repeated three times. Representative images are presented, and the bar graph represents the mean values for the three different experiments. The data are presented as the mean ± SD of three independent experiments (***P < 0.001).

To gain deeper insight into the role of OVOL2 in EMT, we overexpressed OVOL2 using a lentivirus in NMuMG mouse mammary epithelial cells (Figure 1A) and evaluated its effects on TGF-β-induced EMT. Whereas control cells underwent marked EMT within 48 h of TGF-β1 treatment, OVOL2 overexpressing cells retained their epithelial features and formed tighter clusters (Figure 1B). Accordingly, the expression of the epithelial marker E-cadherin was retained in TGF-β1-treated OVOL2 overexpressing cells but lost in control cells, as determined by using confocal immunofluorescence analysis (Figure 1B) and Western blotting (Figure 1C). In contrast, the TGF-β-induced expression of the mesenchymal markers Vimentin, Fibronectin and N-cadherin was dramatically suppressed by OVOL2 (Supplementary Figure 1A). We further found that forced OVOL2 expression inhibited the TGF-β-induced upregulation of numerous EMT-related transcription factors and mesenchymal genes, such as *Zeb1, Zeb2, Twist1, Twist2, Snai2, Fibronectin* and *Vimentin* (Figure 1D), whereas knockdown of OVOL2 further enhanced TGF-β-induced upregulation of these genes (Supplementary Figure 1B). Next, we investigated other EMT phenotypes in TGF-β1-treated cells. The treatment of NMuMG cells with TGF-β1 resulted in enhanced cell migration (Figure 1E) and invasion (Figure 1F). However, the simultaneous overexpression of OVOL2 in these cells almost completely inhibited these TGF-β-induced EMT phenotypes. It is interesting to observe an EMT phenotype when we solely knocked down OVOL2 expression in NMuMG cells, as manifested by increased Vimentin expression and decreased E-cadherin expression (Supplementary Figure 1C).

In order to extend our findings to other cell types, we performed additional experiments. It has been reported that EpH4 murine epithelial cells stably transfected with the H-Ras oncogene (EpRas) could be transformed and induced to undergo EMT upon addition of TGF-β1 [26]. Therefore, we conducted a new assay in this context, and the results indicated that the E-cadherin expression was retained in TGF-β1-treated OVOL2 overexpressing EpRas cells but lost in TGF-β1-treated control EpRas cells, whereas the TGF-β-induced Vimentin expression was greatly suppressed by OVOL2 (Supplementary Figure 2A). We also utilized mesenchymal-like MDA-MB-231 human breast cancer cells, in which TGF-β signaling is aberrantly activated, to determine whether OVOL2 also inhibits EMT phenotypes in MDA-MB-231 cells. As shown in Supplementary Figure 2B, OVOL2 overexpression suppressed the TGF-β signaling-mediated activation of the numerous EMT-related transcription factors investigated in the experiments presented in Figure 1D. OVOL2 overexpression also induced a change in MDA-MB-231 cells from a mesenchymal phenotype to an epithelial phenotype, as manifested by increased expression of the epithelial marker E-cadherin concomitant with the down-regulation of the mesenchymal marker Vimentin, as determined by Western blotting (Supplementary Figure 2C) and confocal immunofluorescence analysis (Supplementary Figure 2D). In addition, the overexpression of OVOL2 resulted in decreased cell migration and invasion, as determined by wound-healing (Supplementary Figure 2E) and Transwell invasion assays (Supplementary Figure 2F).

**Figure 2:**
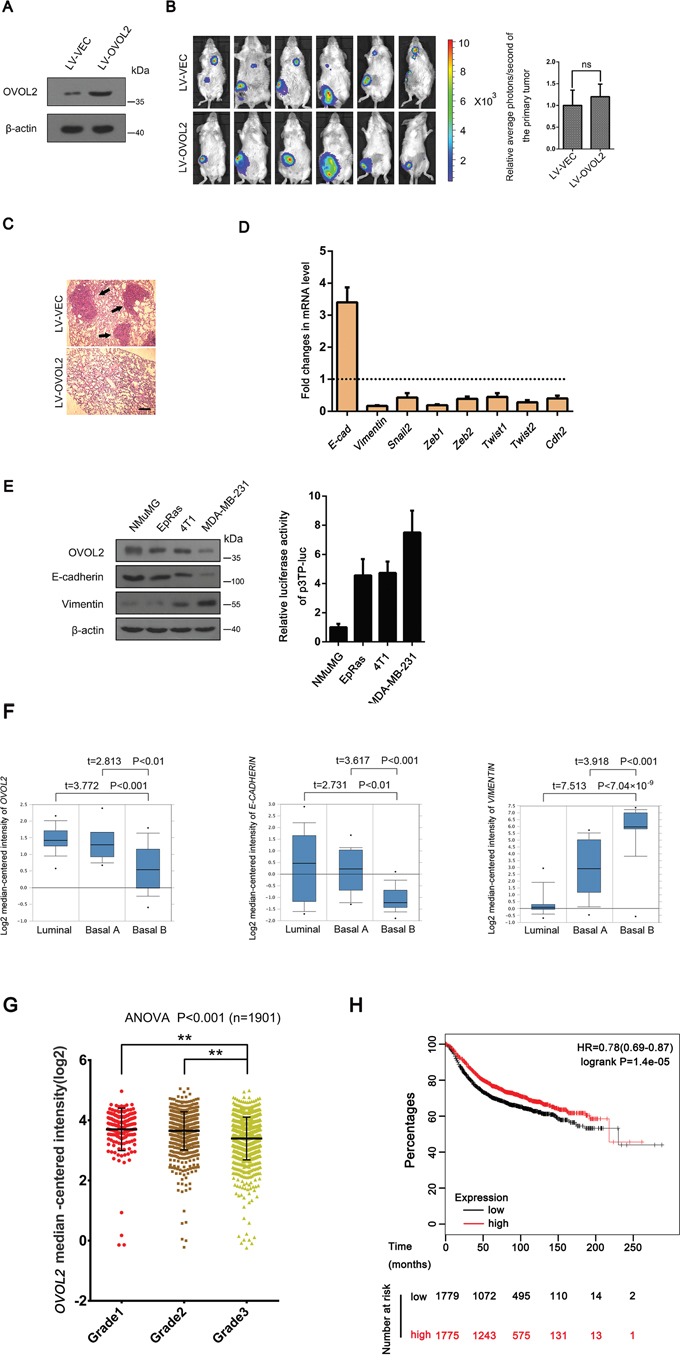
OVOL2 is a candidate EMT suppressor during mammary tumor metastasis **(A)** Western blotting was used to detect the OVOL2 protein levels in 4T1 cells infected with lentiviruses expressing a vector control (LV-VEC) and OVOL2(LV-OVOL2). **(B)** Left, bioluminescence imaging of lung-metastatic breast cancer 4T1 cells at 5 weeks post implantation (5 × 10^5^ cells by orthotopic injection in the fourth mammary gland of BALB/c mice) demonstrating the effects of OVOL2 on the metastatic ability of 4T1 cells. Right, effects of OVOL2 overexpression on the primary tumors. Luciferase expression is depicted as region of interest (ROI-photons/s) in mice orthotropic implantation tumors. Data are the means ± SD, n=6 (ns, not significant). **(C)** Histological examination results for metastatic tumors in the lung (black arrows indicated). Scale bar, 400 μm. **(D)** Quantitative real-time PCR was used to measure the mRNA levels of EMT-related genes in control and OVOL2 overexpressing primary tumors. The real-time PCR values were normalized to the housekeeping gene *Gapdh*. The experiments were performed in technical triplicate. The data are presented as the mean ± SD. **(E)** Western blotting was used to detect the protein levels of OVOL2, E-cadherin and Vimentin in NMuMG, EpRas, 4T1 and MDA-MB-231 cells, and p3TP-luc was transfected into NMuMG, EpRas, 4T1 and MDA-MB-231 cells to measure the basic TGF-β signaling activity. Data are expressed as the mean ± SD of three independent experiments. **(F)** *OVOL2* is downregulated in cell lines that have undergone EMT. *OVOL2* (left) and *E-CADHERIN* (middle) are downregulated, whereas *VIMENTIN* (right) is upregulated in the “basal-B” subclass of breast cancer cell lines reported by Neve and colleagues (Oncomine database). Note, this is the only cohort that divides all the breast cancer cell lines into luminal, Basal A and basal B groups. **(G)** OVOL2 mRNA decreased with human breast cancer progression from grade 2 to grade 3. The data were obtained from the Curtis breast dataset of the Oncomine database and are presented as the mean ± SEM. t-test, **P< 0.01. **(H)** Kaplan–Meier survival curve for relapse-free survival from the KM plotter database. All the patients were separated from middle and defined as high and low OVOL2 expression groups. Proportion of relapse-free cases at different time after surgery was shown. Note, the cases of low OVOL2 expression group (1779) are more than the high OVOL2 expression group (1775), because the OVOL2 expression levels in 4 cases at the middle position are the same.

To further study the effects of OVOL2 on TGF-β signaling and tumor metastasis *in vivo*, an orthotopically implanted 4T1 mouse mammary tumor model was used. We chose this model because 4T1 cells express high levels of TGF-β ligands and receptors and easily metastasize to the lungs in mice [36]. Luciferase-expressing 4T1 cells were infected with a lentivirus expressing OVOL2 or a vector control and were injected into the mammary glands of virgin 6-week-old BALB/c mice. Then, a bioluminescence imaging examination was performed 5 weeks after injection. Bioluminescence imaging results showed that when the tumors in all of 6 control mice exhibited successful lung metastasis, the forced expression of OVOL2 (Figure 2A) completely suppressed this metastasis, whereas the size of the primary tumor was not significantly affected after 5 weeks (Figure 2B). Histological examination of the lung tissue verified these results (Figure 2C). We also confirmed that OVOL2 overexpression had the same effects on the primary tumors as on NMuMG cells and MDA-MB-231 cells with respect to EMT-related gene expression (Figure 2D).

Next, we determined the relationship among the OVOL2 expression, EMT phenotype and the status of TGF-β signaling activation in the above cell types we used. As shown in Figure 2E, OVOL2 expression positively correlated with the E-cadherin expression, but negatively correlated with the Vimentin expression and the TGF-β output levels in these cell lines. Finally, we assessed data from the Neve cell line cohort in the Oncomine database, in which 51 breast cancer cell lines have been classified based on gene expression profiles. One particular subclass (basal-B) was characterized by a mesenchymal profile [36]. *OVOL2* was significantly downregulated, specifically in basal-B cell lines (Supplementary Figure 3A, Figure 2F, left). Accordingly, *E-CADHERIN* exhibited downregulation and *VIMENTIN* exhibited upregulation in this subclass (Figure 2F, middle and right). Consistent with these results from breast cancer cell lines, the data from the Curtis breast cohort which contains the largest number of breast cancer cases in the database, demonstrated that the mRNA expression of OVOL2 significantly decreased with the progression of human breast cancer from grade 2 to grade 3 (Figure 2G), suggesting a strong association between OVOL2 expression and tumor progression. Moreover, we investigated the prognostic value of OVOL2 in breast cancer patients using “the Kaplan-Meier plotter” (KM plotter) database, which contains updated gene expression data and survival information from a total of 3,554 breast cancer patients. When all the patients were separated from middle and defined as high and low OVOL2 expression groups, the results revealed that patients with high OVOL2 expression experienced longer relapse-free survival compared with patients with low OVOL2 expression, with a 22% decrease in the risk of recurrence (Figure 2H), although the overall survival was not significantly different. Taken together, the above data suggest a functional role for OVOL2 in the inhibition of TGF-β-induced EMT and breast tumor metastasis.

### OVOL2 upregulates Smad7 and downregulates Smad4 to inhibit the TGF-β signaling pathway

**Figure 3:**
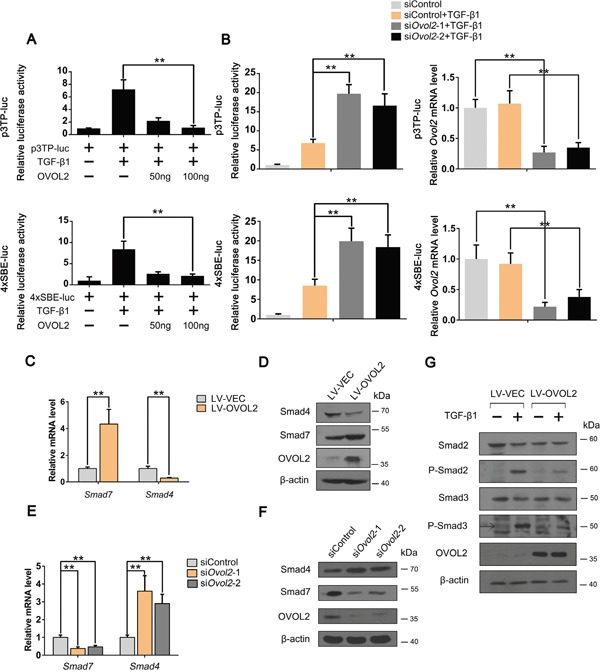
OVOL2 upregulates Smad7 and downregulates Smad4 to inhibit the TGF-β signaling pathway **(A)** Effects of OVOL2 overexpression on TGF-β signaling, as determined by using p3TP-luc (top) and 4xSBE-luc (bottom) luciferase reporters in NMuMG cells. The data are expressed as the mean ± SD of three independent experiments. **P< 0.01. **(B)** Effects of Ovol2 knockdown on TGF-β signaling, as determined by using p3TP-luc (top) and 4xSBE-luc (bottom) luciferase reporters in NMuMG cells. The knockdown efficiency of the endogenous Ovol2 mRNA level is shown in the right panel. The data are expressed as the mean ± SD of three independent experiments. **P< 0.01. **(C)** Quantitative real-time PCR was performed to measure the mRNA levels of Smad4 and Smad7 in control and OVOL2 overexpressing 4T1 cells. The real-time PCR values were normalized to the housekeeping gene *Gapdh*. The experiments were performed three times with real-time PCR performed in technical triplicate. Data are presented as the mean ±SD. **P < 0.01, as indicated by Student's t test. **(D)** Western blotting was performed to detect the protein levels of OVOL2, Smad4 and Smad7 in control and OVOL2 overexpressing 4T1 cells. **(E)** Quantitative real-time PCR was performed to measure the mRNA levels of Smad4 and Smad7 in 4T1 cells transfected with control or *Ovol2* siRNA. The experiments were performed three times, each with real-time PCR performed in technical triplicate, and the data are presented as the mean ± SD. **P < 0.01, as indicated by Student's t test. **(F)** Western blotting was performed to detect the protein levels of Ovol2, Smad4 and Smad7 in 4T1 cells transfected with control or *Ovol2* siRNA. **(G)** Western blotting was performed to detect the protein levels of Smad2, Smad3, P-Smad2 and P-Smad3 in control and OVOL2 overexpressing NMuMG cells.

Because OVOL2 inhibits TGF-β-induced EMT and tumor metastasis, we predicted that TGF-β signaling is directly repressed by OVOL2. To test this hypothesis, we assessed TGF-β/Smad activity by using p3TP-luc and 4xSBE-luc reporter plasmids in NMuMG cells. As shown in Figure 3A, OVOL2 overexpression repressed the transcriptional activation of the TGF-β/Smad-dependent reporters 4xSBE-luc and p3TP-luc by TGF-β1 in a dose-dependent manner. In contrast, when we depleted the endogenous OVOL2 protein using siRNAs against mouse Ovol2, the luciferase assay revealed significantly increased TGF-β/Smad activity (Figure 3B). Notably, when we transfected the empty luciferase vector or p3TP-luc/4xSBE-luc into MDA-MB-231 cells and performed a luciferase assay, we found that the reporters of either p3TP-luc or 4xSBE-luc were significantly activated, which suggests that endogenous TGF-β/Smad signaling is active in MDA-MB-231 cells. However, when OVOL2 was overexpressed, the reporters of either 4xSBE-luc or p3TP-luc were dramatically suppressed (Supplementary Figure 2G), which supports the role of OVOL2 in the inhibition of TGF-β-induced EMT in MDA-MB-231 cells.

To map the position in the TGF-β signaling pathway at which OVOL2 acts, we performed several experiments. First, we overexpressed OVOL2 in 4T1 cells and performed a PCR array assay to detect the expression of all TGF-β/Smad-related components (Supplementary Table 4). Notably, within the core components of the TGF-β/Smad signaling cascade, the co-Smad Smad4 was the most strongly downregulated, whereas I-Smad Smad7 was the most strongly upregulated in 4T1 cells overexpressing OVOL2. Quantitative polymerase chain reaction (qPCR) and Western blotting analyses confirmed these results in cells in which OVOL2 was overexpressed (Figure 3C, 3D) or knocked down (Figure 3E, 3F). Given that OVOL2 acts as a transcriptional repressor, we deduced that OVOL2 activates Smad7 expression via an indirect mechanism. Smad7 recruits the E3 ubiquitin ligases Smurf1 and Smurf2 to activated TGF-β receptors, leading to their degradation via the ubiquitin proteasome system [37–39]. Moreover, Smad7 has been implicated in the association with activated type I receptors and competition with R-Smads for receptor binding [19, 20]. Consequently, the phosphorylation of Smad2 and Smad3 will be inhibited by elevated Smad7 expression. As shown in Figure 3G, the levels of TGF-β-induced phosphorylated Smad2 and Smad3 were both inhibited upon OVOL2 overexpression. Collectively, these results revealed that OVOL2 upregulates Smad7 and downregulates Smad4 to inhibit the TGF-β signaling pathway.

### Smad4 is a novel direct downstream target gene of OVOL2

**Figure 4:**
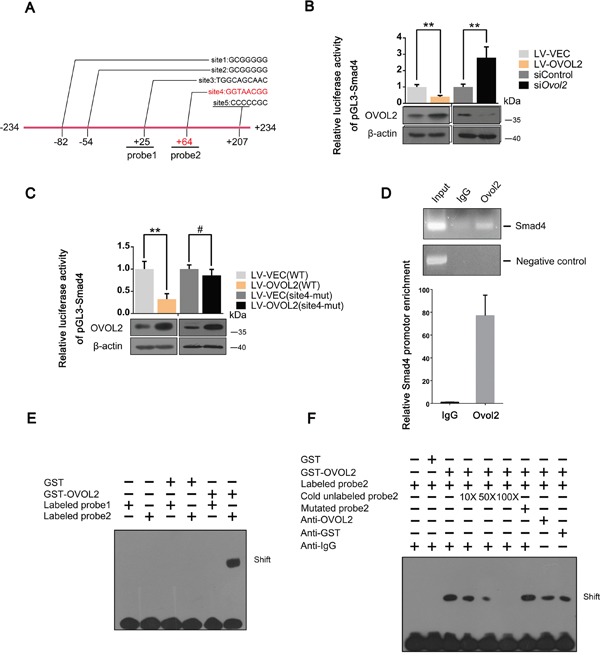
Smad4 is a novel direct downstream target gene of OVOL2 **(A)** Schematic depiction of the Smad4 promoter with several conserved putative Ovol2 binding sites indicated. Sites 1,2,3,5 represent putative binding sites according to classical yet controversial binding sites, while site 4 represents the *bona fide* site. **(B)** Effects of OVOL2 overexpression or Ovol2 knockdown on Smad4 promoter activity, as determined by using the pGL3-basic luciferase reporter cassette in 4T1 cells. The data are expressed as the mean ± SD of three independent experiments. **P< 0.01. **(C)** Effects of OVOL2 overexpression on wild-type Smad4 promoter and mutated Smad4 promoter activities, as determined by using the pGL3-basic luciferase reporter cassette in 4T1 cells. Data are expressed as the mean ± SD of three independent experiments. **P< 0.01, #P > 0.05. **(D)** ChIP assay of endogenous Ovol2 for the Smad4 promoter using semi-quantitative RT-PCR (top) and quantitative real-time PCR (bottom) analyses. A region 10 kb upstream of the Smad4 promoter was used as a negative control. For the RT-PCR analysis, a representative experiment is presented. For the quantitative real-time PCR analysis, the data are expressed as the mean ± SD of three independent experiments. **(E)** Comparison of the binding affinity of GST-OVOL2 for probe1 and probe2 (probe1 was designed according to the putative OVOL2 binding site 3, and probe2 was designed according to the putative OVOL2 binding site 4) using an EMSA assay. **(F)** Further analysis of the binding affinity of GST-OVOL2 to probe2 using an EMSA assay.

Given that Smad4 is negatively regulated by OVOL2, we asked whether Smad4 is a direct OVOL2 target gene. To test this hypothesis, we examined the genomic sequence within a 10-kb window centered on the transcriptional start site (TSS) of mouse Smad4. The genomic sequence that extends from -234 bp upstream to +234 bp downstream of the TSS contains four putative OVOL2 binding sites (sites 1, 2, 3, and 5), which are conserved between humans and mice (Figure 4A). First, we performed a luciferase assay by cloning this fragment into the PGL3-basic luciferase reporter cassette. The fragment was sufficient to inhibit luciferase reporter activity when OVOL2 was overexpressed in 4T1 cells and to enhance luciferase reporter activity when OVOL2 expression was knocked down in 4T1 cells (Figure 4B), indicating that Smad4 is indeed a downstream target of OVOL2. The sequence of the consensus OVOL2 binding site is controversial and includes G(G/T/C)GGGGG [40] (sites 1,2 and 5) and A(A/T) (A/T)(C/A)(T/C)GTTA(T/A) [32] (site 3). However, when all 4 putative OVOL2 binding sites were mutated, no significant change in OVOL2 inhibition was observed (data not shown). Therefore, we hypothesized that OVOL2 utilizes a novel binding site to downregulate Smad4 expression. To address this question, we re-examined this fragment and found a conserved sequence (GGTAACGG (site 4)) that strongly resembles the *Drosophila* OVO consensus binding site AGTAACNG [41] at +64 bp. The mutation of site 4 resulted in diminished inhibition of Smad4 promoter activity by OVOL2 (Figure 4C). In a chromatin immunoprecipitation (ChIP) assay, OVOL2 occupies the endogenous Smad4 promoter at this novel site in 4T1 cells (Figure 4D). Given that site 4 is very close to site 3, the ChIP assay cannot distinguish between these sites. Thus, we performed an electrophoretic mobility shift assay (EMSA) using 2 oligonucleotides (oligo1 and oligo2) that span sites 3 and 4, respectively (Figure 4A). EMSA results revealed that recombinant OVOL2 bound to oligo2, which contains site 4, but not to oligo1, suggesting that OVOL2 indeed binds to the oligo that contains GGTAACGG (Figure 4E). Whereas the presence of unlabeled oligo2 completely abolished the gel-shift band, oligos containing mutations in the GGTAACGG sequence failed to compete for binding (Figure 4F). Together, these results identify a *bona fide* OVOL2 binding site in the Smad4 promoter.

### OVOL2 associates directly with Smad4

**Figure 5:**
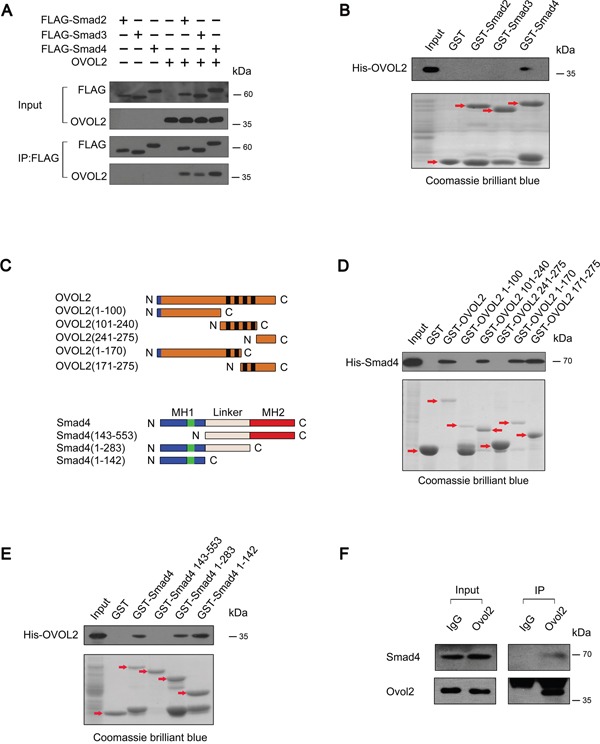
OVOL2 associates directly with Smad4 **(A)** HEK293T cells were transfected with the indicated combinations of FLAG-tagged Smad2/Smad3/Smad4 and untagged OVOL2. The cell lysates were immunoprecipitated with an anti-FLAG antibody followed by immunoblotting to detect the protein levels of Smad2/3/4 and immunoprecipitated OVOL2. **(B)** *In vitro* pull-down assay using GST-tagged Smads and His-tagged OVOL2 demonstrated direct binding between OVOL2 and Smad4 but not Smad2 or Smad3; the red arrows indicate target bands. **(C)** Top, schematics of the OVOL2 protein and the various truncated versions of the protein. The blue boxes represent the SNAG domain and the black boxes represent the zinc-finger structure. Bottom, schematics of the Smad4 protein and the various truncated versions of the protein. MH1 contains the DNA binding domain. The green boxes represent the NLS. The MH2 is responsible for receptor recognition and oligomerization with other Smads. N: N-terminal, C: C-terminal. **(D)** *In vitro* pull-down assay using GST-tagged OVOL2/OVOL2 truncates and His-tagged Smad4 demonstrated direct binding between Smad4 and OVOL2 at either the first two or the last two zinc fingers; the red arrows indicate target bands. **(E)** *In vitro* pull-down assay using GST-tagged Smad4/Smad4 truncates and His-tagged OVOL2 demonstrated direct binding between OVOL2 and Smad4 at its MH1 domain; the red arrows indicate target bands. **(F)** Endogenous co-IP experiments between Ovol2 and Smad4 from nuclear extracts of non-transfected 4T1 cells.

We asked whether OVOL2 also regulates TGF-β signaling via a protein-protein interaction mechanism. To verify this hypothesis, we detected the association between OVOL2 and Smad2/3 or Smad4 via a co-immunoprecipitation (co-IP) assay of HEK293T cells transfected with OVOL2 and FLAG-tagged Smad2/3 or Smad4. As shown in Figure 5A, OVOL2 interacted with Smad2, Smad3, and Smad4. Next, an *in vitro* pull-down assay was performed to examine whether OVOL2 associates directly with these Smads. The results indicated that OVOL2 only associates directly with Smad4 (Figure 5B). We also created various truncated versions of the two constructs to assess which region is responsible for the binding (Figure 5C). The OVOL2 protein contains an N-terminal SNAG motif and a central region that contains four C2H2 zinc fingers. Our results revealed that OVOL2 directly associates with Smad4 and that either the first two or the last two zinc fingers are sufficient for binding (Figure 5D). Similarly, we also mapped the Smad4 interaction domain to its DNA-binding MH1 domain (Figure 5E). Finally, we investigated whether the endogenous proteins interact by performing immunoprecipitation experiments using nuclear extracts from non-transfected 4T1 cells. As shown in Figure 5F, the immunoprecipitation of OVOL2 pulled down Smad4, suggesting that OVOL2 is normally present in a complex with Smad4 in cells.

### OVOL2 interferes with complex formation between Smad4 and Smad2/3 and inhibits the binding between Smad4 and target DNA

**Figure 6:**
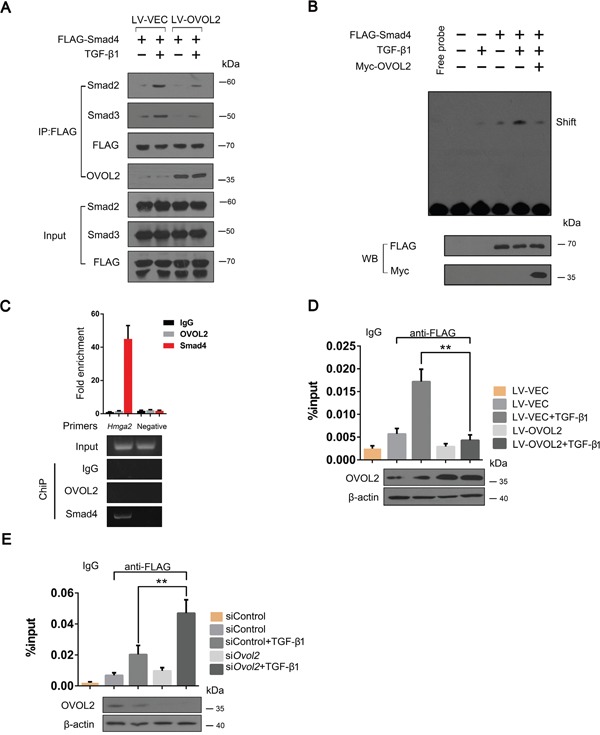
OVOL2 interferes with complex formation between Smad4 and Smad2/3 and blocks the binding between Smad4 and target DNA **(A)** NMuMG cells treated with lentiviruses expressing a vector control and OVOL2 were transfected with FLAG-tagged Smad4 and treated with or without TGF-β1 for 48 h. The cell lysates were subjected to anti-FLAG immunoprecipitation, and endogenous co-immunoprecipitating Smad2/3 and OVOL2 were detected by immunoblotting with appropriate antibodies. **(B)** Effect of OVOL2 on the binding efficiency of Smad4 for the 4xSBE probe using an EMSA assay in 4T1 cells. The 4T1 cells were transfected with FLAG-tagged Smad4 or Myc-OVOL2 and treated with or without TGF-β1 for 48 h. The cell lysates were subjected to EMSA assay by using 4xSBE probe. **(C)** Chromatin immunoprecipitation (ChIP) assay of OVOL2 or Smad4 at the Hmga2 promoter in 4T1 cells using semi-quantitative PCR (bottom) and qPCR (top) analyses with the indicated primers and antibodies. A region 10 kb upstream of the Hmga2 promoter was used as a negative control. For the RT-PCR analysis, a representative experiment is presented. For the quantitative real-time PCR analysis, the data are expressed as the mean ± SD of three independent experiments. **(D, E)** Effect of OVOL2 overexpression **(D)** or knockdown **(E)** on the binding efficiency of Smad4 to the Hmga2 promoter in 4T1 cells. The experiments were performed three times, each with real-time PCR performed in technical triplicate, and the data are presented as the mean ± SD. **P < 0.01, as indicated by Student's t test.

Because OVOL2 inhibits the phosphorylation of Smad2 and Smad3 (Figure 3G), we predicted that OVOL2 interferes with complex formation between Smad4 and Smad2/3. To investigate this hypothesis, we expressed FLAG-Smad4 exogenously in NMuMG cells and performed a co-IP assay by using an anti-FLAG antibody, reasoning that endogenous Smad4 is also downregulated by OVOL2. As shown in Figure 6A, when the cells were stimulated with TGF-β1, FLAG-Smad4 pulled down large amounts of Smad2 or Smad3 in NMuMG cells. However, in cells overexpressing OVOL2, the associations between FLAG-Smad4 and Smad2 or Smad3 were significantly inhibited. Because OVOL2 interacts with Smad4 via its DNA-binding domain (MH1)-containing N-terminal region, it is possible that OVOL2 blocks the binding between Smad4 and target DNA. Because SBE (Smad-binding DNA element) sequence can bind with Smad4, we therefore synthesized a 4xSBE probe and performed an EMSA in 4T1 cells. As shown in Figure 6B, the solely stimulation by TGF-β1 or overexpression of Smad4 resulted in week binding between Smad4 and the 4xSBE probe, and the binding was significantly enhanced when cells were treated by both. However, additional transient overexpression of Myc-OVOL2 in the above cells resulted in dramatically reduced binding of Smad4 with the 4xSBE probe. To address whether OVOL2 also affects the binding of Smad4 to the target chromatin, we used Hmga2 to perform a ChIP assay because Hmga2 is a direct target of TGF-β-Smad and induces the expression of Snail, Slug, and Twist [42, 43]. We firstly demonstrated that Smad4 but not OVOL2 occupied Hmga2 promoter region (Figure 6C). Next, we performed the ChIP assay using FLAG-Smad4-overexpressing 4T1 cells, and the results showed that the overexpression of OVOL2 led to reduced occupancy of the Hmga2 promoter by FLAG-Smad4 (Figure 6D). In contrast, the knockdown of OVOL2 expression in 4T1 cells resulted in increased occupancy by FLAG-Smad4 (Figure 6E). Together, these results provide compelling evidence that OVOL2 interferes with complex formation between Smad4 and Smad2/3 and blocks the binding between Smad4 and target DNA.

## DISCUSSION

OVOL2 has been implicated in the regulation of EMT process in the mouse mammary gland and human breast cancer cells through the transcriptional inhibition of ZEB1 [33, 35]. However, the underlying molecular mechanism and the association between OVOL2 and tumor progression has not been systematically elucidated. In current study, we establish the relationship between OVOL2 expression and EMT phenotype or tumor progression by checking breast cancer cell lines and patients cohorts in the public databases. Moreover, we identify TGF-β signaling as another target that OVOL2 antagonizes, suggesting that targeting signaling pathways may be one general mechanism underlying OVOL2's anti-cancer functions. Our experiments indicate that OVOL2 inhibits TGF-β signaling at multiple levels, including the inhibition of Smad4 mRNA expression and the induction of Smad7 mRNA expression. In addition, OVOL2 blocks the binding between Smad4 and target DNA, and interferes with complex formation between Smad4 and Smad2/3. These results suggest that OVOL2 is involved in the regulation of the core components of TGF-β signaling during breast cancer progression. Particularly, our results demonstrate that OVOL2 inhibits the binding between Smad4 and promoter DNA, which represents the very bottom of the signaling cascade. Hence, the other functions of OVOL2 appear to be dispensable. However, as protein functions are typically regulated dynamically, it is possible that OVOL2 exerts these functions in a sequential or random manner, resulting in a strong suppression of TGF-β signaling output.

It is noted that the OVOL proteins have been identified as transcription inhibitors that repress gene expression by binding to DNA sequences in the promoter region [44]. Consistently, recent studies of OVOL2 revealed its function in the regulation of EMT through the transcriptional inhibition of ZEB1 mRNA expression [33, 35]. Our observation in this study also demonstrates an EMT phenotype when we solely knocked down OVOL2 expression in NMuMG cells. We deduce that OVOL2 exerts this function mainly through ZEB1. However, the current study shows that in addition to inhibiting Smad4 expression directly, OVOL2 inhibits TGF-β signaling activity by binding to the Smad4 protein. These observations suggest that OVOL2 may adopt alternative mechanisms, which may act on the furthest upstream of EMT controlling cascade to regulate EMT in both the mouse and human mammary tumor cells.

Numerous studies have revealed the role of TGF-β signaling in EMT in cancers; however, the regulation of this signaling process during EMT has not been completely defined. Since somatic mutations of TGF-β signaling components are rare in many cancers, including breast cancer [15, 16, 45, 46], the TGF-β output level is primarily controlled by Smads interaction partners. Accumulating evidence has demonstrated that the tumor suppressor functions of Smads are compromised by oncogene products, such as c-Ski, Bcl6, c-Myc, Evi-1 and STAT3, through direct Smad–oncoprotein interactions during carcinogenesis [47–50]. Our observations in this study revealed a similar mechanism for the regulation of the tumor-promoting functions of Smads during cancer progression. Since OVOL2 expression in breast cancer specimens progressively declines due to its promoter methylation (unpublished data), we reason that OVOL2 might contribute to TGF-β inhibition in early stage of cancers. Along with the progression of breast cancer, OVOL2 expression is downregulated, resulting in its inability to inhibit TGF-β signaling. Therefore, TGF-β signaling releases the suppression by OVOL2; once the TGF-β output level reaches a threshold level that is sufficient to induce the EMT, cancer invasion and metastasis occur.

In summary, we have identified a novel mechanism that controls TGF-β signaling output levels *in vitro* and *in vivo*, which sheds new light on the regulation of EMT in cancers. The modulation of these molecular processes may represent a strategy for inhibiting breast cancer invasion by restoring OVOL2 expression.

## MATERIALS AND METHODS

### Cell culture

HEK 293T, NMuMG, EpH4, 4T1 and MDA-MB-231 cells were obtained from ATCC (American Type Culture Collection, Manassas, VA, USA). These cell lines were cultured in DMEM containing 10% fetal bovine serum supplemented with 100 units/ml of penicillin and 100 μg/ml of streptomycin. The cells were incubated at 37°C in a humidified atmosphere of 5% CO_2_.

### Generation of cDNA-expressing lentivirus

For overexpression of mus musculus OVOL2, the cDNA was cloned under the control of the EF-1α promoter in the lentiviral vector of pLV-CS2.0. Generation of lentivirus vector was performed by co-transfecting pLV-CS2.0 carrying the expression cassette with helper plasmids pVSV-G and pHR into HEK293T cells using Lipofectamine 2000 (Invitrogen). The viral supernatant was collected 48h after transfection. Cells at 50% to 70% confluence were infected with viral supernatants containing 10 μg/ml Polybrene for 24 h, after which fresh medium was added to the infected cells.

### RNA extraction and real-time PCR

Total RNA was isolated from the cells using the Trizol reagent (Invitrogen, Carlsbad, CA, USA), in accordance with the manufacturer's instructions. cDNA was randomly primed from 2.0 μg of total RNA using the HiFi-MMLV cDNA Kit (Cwbio, Beijing, China). Real-time PCR was performed using the Platinum SYBR Green qPCR SuperMix (Invitrogen, Carlsbad, CA, USA) in the CFX96 Real Time System. All real-time PCR assays were performed in technical triplicate in at least three independent experiments using three different samples. In addition, all mRNA quantification data were normalized to the house-keeping gene. The specific primer sequences are described in Supplementary Table 1.

### Chromatin immunoprecipitation

The cells were plated in 10-cm dishes and grown to approximately 80%–90% confluency. Then, the cells were fixed with 1% paraformaldehyde and lysed with FA Lysis Buffer. The lysates were sonicated to reduce the DNA lengths to between 500 and 1000 bp. The lysates were immunoprecipitated with specific antibody. The DNA product was analyzed using real-time PCR and semi-quantitative PCR. The PCR products were separated by electrophoresis on 2% low-melt agarose gels and visualized with GoldView. The primer sequences are presented in Supplementary Table 2.

### Electrophoretic mobility shift assay (EMSA)

EMSA was performed according to the protocol provided with the EMSA/Gel-Shift kit (Beyotime Biotechnology, Shanghai, China). In parallel, to determine binding specificity, competition experiments were performed with unlabeled wild-type or mutated probe. DNA binding was performed in a 10 μL reaction volume containing EMSA/Gel-Shift Binding Buffer (Beyotime, Shanghai, China), 2 nM labeled DNA fragment and 5 nM protein. After incubation at 25°C for 30 min, the products were loaded onto a native 4 % (W/V) polyacrylamide gel and electrophoresed in 0.5 × TBE buffer for about 1.5 h at 100 V. The probe sequences are presented in Supplementary Table 3.

### Luciferase assay

Cells were seeded in 24-well plates 24 h before transfection. The following day, the cells were co-transfected with the indicated combination of expression plasmids (β-galactosidase, 4xSBE-luc, 3TP-luc) at 50 to 60% confluency using a calcium-phosphate method and treated with or without 5 ng/ml of TGF-β1. Then, the cells were harvested 24 h post-transfection and processed for luciferase and β-galactosidase assays. The data were normalized to β-galactosidase levels.

### Immunoprecipitation, western blotting and antibodies

The transfected cells were lysed in lysis buffer (20 mMTris-HCl, pH 7.5, 150 mMNaCl, 1 mM EDTA, pH 8.0, 1 mM EGTA, pH 8.0, and 1% Triton X-100) supplemented with protease inhibitors, including phenylmethyl-sulfonyl fluoride (PMSF) and cocktail (Sigma-Aldrich, St Louis, MO, USA). The lysates were then pre-cleared with protein A/G beads for 1 h at 4°C with gentle agitation. The extracts were then incubated with 2 μg of the corresponding specific antibody and 20 μl of fresh protein A/G beads at 4°C with agitation overnight. The collected protein complexes were washed three times with washing buffer, mixed with 5X SDS loading buffer and boiled for 10 minutes. The co-precipitates or whole-cell extracts were resolved by sodium dodecyl sulfate polyacrylamide gel electrophoresis (SDS-PAGE) and blotted onto polyvinylidenedifluoride (PVDF) membranes (Millipore, Bedford, MA, USA). The membranes were immunoblotted with the indicated antibodies and developed using an enhanced chemiluminescence (ECL) detection system. Anti-OVOL2 (ab101580; 1:500) and anti-Smad7 (ab90086; 1:1000) were purchased from Abcam (Cambridge, England), anti-HA (H9658; 1:5000), anti-FLAG (F7425; 1:5000), anti-Myc (C3956; 1:5000), anti-His (SAB4600371; 1:2000) and anti-β-actin (A1978; 1:5000) were purchased from Sigma-Aldrich (St Louis, MO, USA), anti-Smad2 (3122S; 1:1000), anti-Smad3 (9523S; 1:1000), anti-Smad4 (9515S; 1:1000), anti-P-Smad2 (3101S; 1:1000) and anti-P-Smad3 (9520S; 1:1000) were purchased from Cell Signaling Technology (Danvers, MA, USA).

### PCR array

The RT^2^ Profiler PCR Array (Qiagen, Hilden, Germany) was used to profile the expression of 84 key genes involved in TGF-β/Smad-related components.

Synthesis of the cDNA was performed with the RT^2^ first strand kit (Siegen) according to the manufacturer's instructions at 42°C for 15 min with a 5-min deactivation step at 95°C in a BioRad CFX96 Real-Time System C1000 Thermal Cycler (Biorad, Munic, Germany).

The RT^2^ SYBR green master mix (Qiagen) (1350 μl per 96-well plate) was mixed with 1248 μl RNase free water and 102 μl cDNA synthesis reaction template, and 25 μl PCR components were added to each well of the array. Quantitative real time polymerase chain reaction (qRT-PCR) was performed in accordance with the recommendations of the manufacturer. Cycling and detection were done in a BioRad CFX96 Real-Time System C1000 Thermal Cycler (Biorad, Munic, Germany).

### Statistical analysis

The data were analyzed using the GraphPad Prism 5.0 (San Diego, CA, USA) software. All data are presented as the mean ± standard error of the mean unless specified otherwise. Student's t-test, the Pearson's r test and a one-way analysis of variance (ANOVA) were used to compare data and to calculate P-values. All the statistical tests were two-sided. P<0.05 was considered significant.

### Animal studies

Animal care and handling procedures were performed in accordance with the Guide for the Care and Use of Laboratory Animals, and the animal study protocol was approved by the Institutional Animal Care and Use Committee of Xiamen University (Reference No.XMULAC20150069). Briefly, 6- to 8-week-old female BALB/c mice were supplied by the Xiamen University Laboratory Animal Center, China. All mice were kept under specific pathogen-free conditions and had free access to a standard diet and drinking water. Cells were injected orthotopically into one of the fourth mammary gland of the mice. The mice underwent bioluminescence imaging 5 weeks after the injection. The lungs were isolated, routinely stained with hematoxylin-eosin (HE) and evaluated under a light microscope. The investigator who performed the bioluminescence imaging assay was blinded to group allocation.

### Cell invasion assay

The *in vitro* invasion assay was performed using BioCoat Matrigel invasion chambers (BD Bioscience, Franklinlakes, New Jersey, USA). The inserts were coated on the inside with 1-2 mg/ml of Matrigel per insert. Cells were placed into the upper chamber in 0.5 ml of serum-free medium. The lower compartment was filled with complete medium. 48 h later, the cells migrated to the lower surface of the filters. Finally, the cells were fixed in ethanol for 5 minutes at room temperature and visualized using a Crystal Violet staining method.

### Immunofluorescence staining

Cells were grown on sterile coverslips. 24 h later, the cells were washed twice with phosphate-buffered saline (PBS), fixed with 4% paraformaldehyde for 10 minutes at room temperature, permeabilized with 0.5% Triton X-100 for 10 minutes, and blocked with 1% bovine serum albumin (BSA) for 30 minutes at 37°C. The coverslips were then stained with primary antibodies at 4°C overnight followed by the appropriate secondary antibodies. DAPI staining served to label the cell nuclei. Images were collected with a Zeiss LSM710 confocal microscope (Oberkochen, Germany).

### Wound-healing assay

Cells were seeded in 6-cm culture dishes in complete DMEM. After treatment with or without TGF-β, the monolayers were scratched with a 200 μl plastic pipette tip to create a uniform wound. Then, the monolayers were washed with PBS and incubated in culture medium supplemented with 10% fetal bovine serum (FBS). The wound margin distances between the two edges of the migrating cell sheets were photographed after scratching using phase-contrast microscopy. All experiments were performed in triplicate.

### Kaplan-Meier survival analysis

Briefly, KaplanMeier (KM) plots were attained using the KMPlotter web-based (kmplot.com/analysis) curator, which surveys public microarray repositories for relapse free and overall survival among patients with breast, lung, ovarian or gastric cancers.

### Glutathione S-transferase (GST) pull-down assay

GST fusion proteins were expressed in the E. coli strain BL21. To purify the GST fusion proteins, the cells were lysed by sonication in lysis buffer, and the resulting lysates were incubated for 1 h at 4°C with glutathione–Sepharose beads. The beads were pelleted by centrifugation and washed with dialysis buffer for subsequent experiments. The nuclear extracts were then incubated with resin-bound proteins by rotating at 4°C for 3 h, washed four times in washing buffer, and separated via SDS-PAGE. The gels were stained with Coomassie Blue to ensure that the proteins used in the assays were of similar purity and to estimate protein loading.

## SUPPLEMENTARY FIGURES AND TABLES




