# Supplementary material for: OVOL2 antagonizes TGF-β signaling to regulate epithelial to mesenchymal transition during mammary tumor metastasis
Source: Oncotarget. 2017 Apr 11;8(24):39401–16. doi: 10.18632/oncotarget.17031 (PMC5503621; doi:10.18632/oncotarget.17031)
Supplement: Supplementary file 1 [file oncotarget-08-39401-s001.pdf]

OVOL2 antagonizes TGF-β signaling to regulate epithelial to mesenchymal transition during mammary tumor metastasis

SUPPLEMENTARY FIGURES AND TABLES

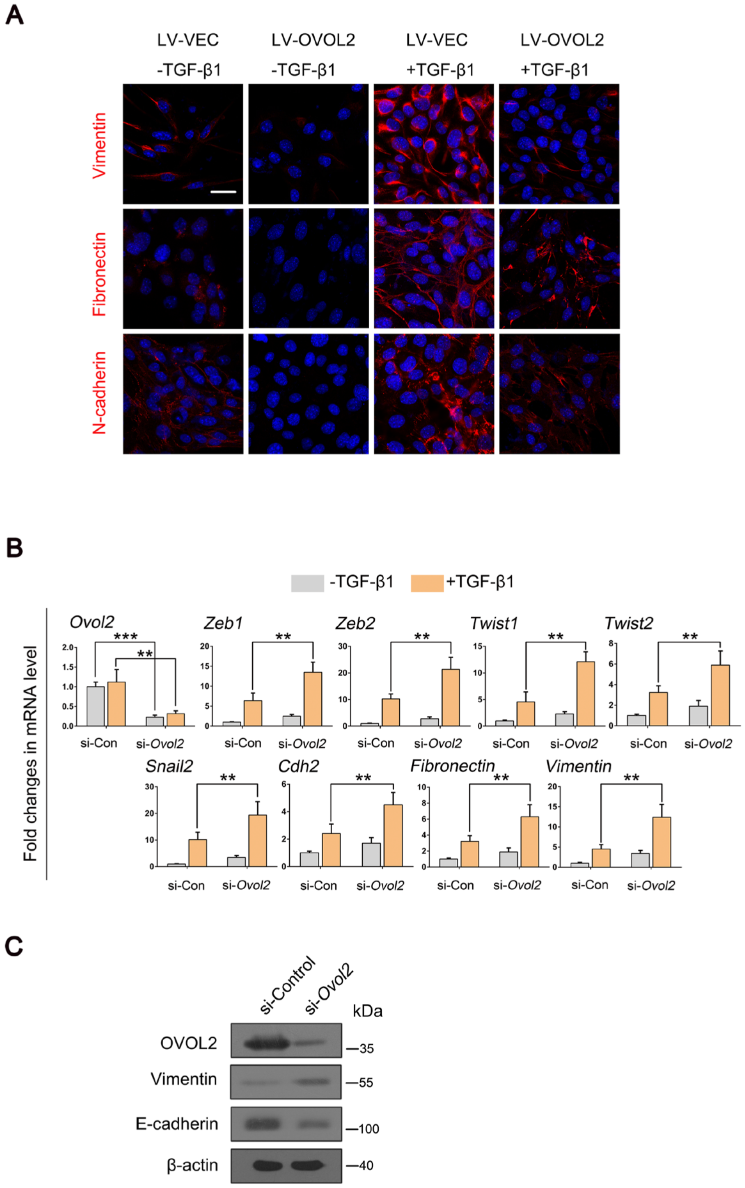

Supplementary Figure 1: OVOL2 inhibits TGF-β-induced EMT during mammary tumor metastasis. (A) Immunofluorescence images of control and OVOL2 overexpressing NMuMG cells, with or without TGF-β1 treatment, stained for N-cadherin, Fibronectin and Vimentin. Scale bars, 20 µm. **(B)** Quantitative real-time PCR was used to measure the mRNA levels of EMT-related genes in si-control and si-*Ovol2* NMuMG cells with or without TGF-β1 (5 ng/ml 48 h) treatment. The real-time PCR values were normalized to the housekeeping gene *Gapdh*. The experiments were performed three times, each with real-time PCR performed in technical triplicate, and the data are presented as the mean ± SD. $^{**}P < 0.01$, $^{***}P < 0.001$, as indicated by Student's t-test. **(C)** The knockdown of OVOL2 expression in NMuMG cells resulted in an EMT phenotype. Western blotting was used to detect the protein levels of OVOL2, E-cadherin and Vimentin in si-control and si-*Ovol2* NMuMG cells.

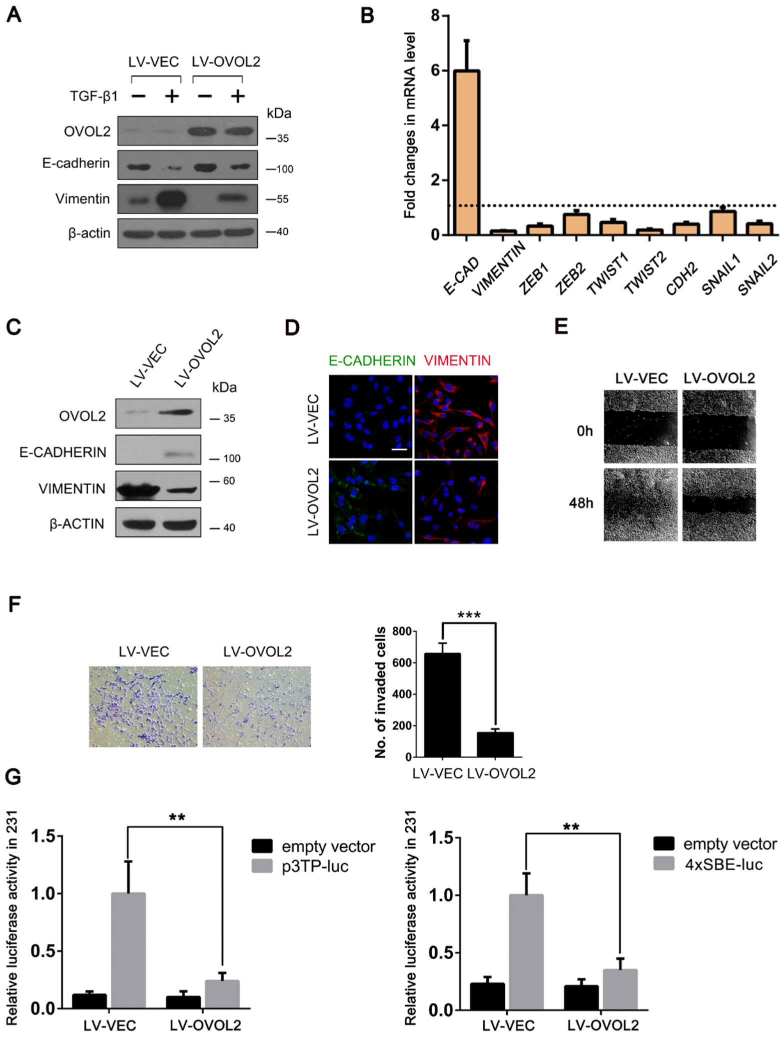

Supplementary Figure 2: OVOL2 inhibits EMT in EpRas cells and human MDA-MB-231 cells. **(A)** Western blotting was used to detect the protein levels of OVOL2, Vimentin and E-cadherin in control and OVOL2 overexpressing EpRas cells (Ras-transformed mouse mammary epithelial cells) with or without TGF-β1 (5 ng/ml 48 h) treatment. **(B)** Quantitative real-time PCR was used to measure the mRNA levels of EMT-related genes in control and OVOL2 overexpressing MDA-MB-231 cells. The real-time PCR values were normalized to the housekeeping gene *GAPDH*. The experiments were performed thrice, each with real-time PCR performed in technical duplicate. Data are presented as the mean ± SD. **(C)** Western blotting was used to detect the protein levels of OVOL2, VIMENTIN and E-CADHERIN in control and OVOL2 overexpressing MDA-MB-231 cells. **(D)** Immunofluorescence images of control and OVOL2 overexpressing MDA-MB-231 cells stained for E-CADHERIN and VIMENTIN. Scale bar, 20 μm. **(E)** The migration ability of the MDA-MB-231 cells described above was assessed using a wound-healing assay. The experiment was performed in triplicate. Representative images are presented. **(F)** The invasive ability of the above MDA-MB-231 cells is presented as the total number of cells that entered the bottom invasion chamber, counted across eight fields. Each sample was measured in triplicate, and each experiment was repeated thrice. Representative images are shown, and the bar graph shows the mean values for the three different experiments. Data are presented as the mean ± SD of three independent experiments (***$P < 0.001$). **(G)** An empty vector and p3TP-luc (left) or 4xSBE-luc (right) were transfected into control and OVOL2 overexpressing MDA-MB-231 cells to measure the effect of OVOL2 on TGF-β signaling. Data are expressed as the mean ± SD of three independent experiments. **$P< 0.01$.

A

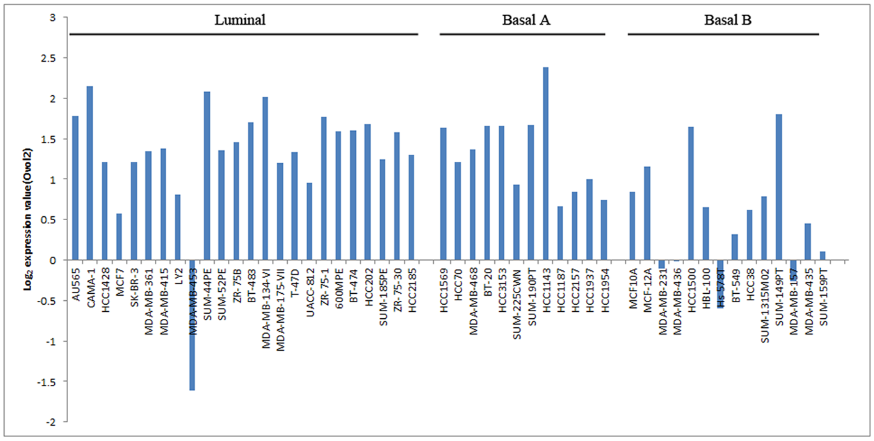

Supplementary Figure 3: OVOL2 is downregulated in cells that have undergone EMT. (A) *OVOL2* is downregulated in the "basal-B" subclass of breast cancer cell lines described by Neve and colleagues (Oncomine database).

Supplementary Table 1: Primers for RT-PCR

Primers for RT-PCR	Sequences (5’-3’)
Cdh2-F	AAGGCATTCAGCACCCACCT
Cdh2-R	AGGCGGGATTCCATTGTCAGA
Twist1-F	AGGCCGGAGACCTAGATGTCATT
Twist1-R	TGCCCCACGCCCTGATTC
Twist2-F	CACGAGCGTCTCAGCTACGC
Twist2-R	TGGGTCCTGGCTTGCGG
Zeb1-F	TGCTGGCAAGACAACGTGAAAG
Zeb1-R	CACAATACGGGCAGGTGAGC
Zeb2-F	TCCGTCCCCAGGTTTGTGTTT
Zeb2-R	AGTTCACCACGTTTTTCCTCCTG
Snai1-F	TAACAAGGAGTACCTCAGCCTGG
Snai1-R	GGTCAGCAAAAGCACGGTTG
Snai2-F	CTGGCTGCTTCAAGGACACATT
Snai2-R	GAAGGTTTTGGAGCAGTTTTTGC
Gapdh-F	CCACTCTTCCACCTTCGATGC
Gapdh-R	GTCTGGGATGGAAATTGTGAGG
Vimentin-F	GAATGACCGCTTTGCCAACTAC
Vimentin-R	GACGTGCCAGAGAAGCATTGTC
Fibronectin-F	GCTGTCTCCACCGACCAACT
Fibronectin-R	GGGGCACCTCTGGGACAAC
Smad4-F	ACACCAACAAGTAACGATGCC
Smad4-R	GCAAAGGTTTCACTTTCCCCA
Smad7-F	CTCCTCCTTTCTCGTCATCC
Smad7-R	CACACACACAACCCAACAAA
CDH2-F	AGATAGCCCGGTTTCATTTGAG
CDH2-R	ATGTTGGGTGAAGGGGTGCT
FIBRONECTIN-F	CACTTCAGTGGGAGACCTCGAG
FIBRONECTIN-R	GGTCCCTCGGAACATCAGAAAC
TWIST1-F	CGGCTCAGCTACGCCTTCT
TWIST1-R	CAATGACATCTAGGTCTCCGGC
TWIST2-F	CATGTCCGCCTCCCACTAGC
TWIST2-R	ATGTGCTCACTCCCGCCAAC
SNAIL1-F	GCTACTGCTGCGCGAATCG
SNAIL1-R	GTAGGGCTGCTGGAAGGTAAACT
SNAIL2-F	TTCGGACCCACACATTACCTTG
SNAIL2-R	ACCTGTCTGCAAATGCTCTGTTG
GAPDH-F	GGAGTCAACGGATTTGGTCGTA
GAPDH-R	GGCAACAATATCCACTTTACCAGAGT
ZEB1-F	AGGCTATAAACGCTTTACCTCTCTG
ZEB1-R	TTACGATTACACCCAGACTGCGT
ZEB 2-F	CGGTAAGAAGCAGCCCGAAC
ZEB 2-R	TTGTTGTGCCAGGGGTGTTC
OVOL2-F	CGGATGAGAAAAGGGCAGACA
OVOL2-R	TGTGGCACTTGAGGTGACGG
VIMENTIN-F	AATGGCTCGTCACCTTCGTG
VIMENTIN-R	CAGATTAGTTTCCCTCAGGTTCAG'

Supplementary Table 2: Primers for ChIP

Primers for ChIP	Sequences (5’-3’)
Smad4-F	GCCAAACCCTGAAATTACCCG
Smad4-R	CCACCCAAA CCGCTCCGT
Hmga2-F	AATAGTAGACAGCGGCGGTT
Hmga2-R	TCCTAGGAAGGCGAAAGAAA

Supplementary Table 3: Probes for EMSA

Probes for EMSA	Sequences (5’-3’)
Labeled probe1-F	CTCGACAAGTTGGCAGCAACAACACGGCCC
Labeled probe1-R	GGGCCGTGTTGTTGCTGCCAACTTGTCGAG
Mut-labeled probe1-F	CTCGACAAGTTGGCAGGGACAACACGGCCC
Mut-labeled probe1-R	GGGCCGTGTTGTCCCTGCCAACTTGTCGAG
Labeled probe2-F	GTCGTCGCCGCTGCGGTAACGGAGCGGTTT
Labeled probe2-R	AAACCGCTCCGTTACCGCAGCGGCGACGAC
Mut-labeled probe2-F	GTCGTCGCCGCTGCGGGGACGGAGCGGTTT
Mut-labeled probe2-R	AAACCGCTCCGTCCCCGCAGCGGCGACGAC

Supplementary Table 4: Fold Difference in TGF-β/BMP4 pathway PCR Array gene expression for OVOL2 overexpressed 4T1 cells related to control 4T1 cells

See Supplementary File 1
