# Supplementary material for: OVOL2 antagonizes TGF-β signaling to regulate epithelial to mesenchymal transition during mammary tumor metastasis
Source: Oncotarget. 2017 Apr 11;8(24):39401–16. doi: 10.18632/oncotarget.17031 (PMC5503621; doi:10.18632/oncotarget.17031)
Supplement: Supplementary file 2 [file oncotarget-08-39401-s002.docx]

**Supplementary Table 4: Fold Difference in TGF-β/BMP4 pathway PCR Array gene expression for OVOL2 overexpressed 4T1 cells related to control 4T1 cells**

|  | Assay ID | Gene Symbol | Fold Difference |
| --- | --- | --- | --- |
| TGF-β Superfamily Ligands | Mm00439683_m1 | [Inha](http://www.ncbi.nlm.nih.gov/entrez/query.fcgi?db=gene&cmd=Retrieve&dopt=Graphics&list_uids=16322) | 1.49 |
|  | Mm00434339_m1 | [Inhba](http://www.ncbi.nlm.nih.gov/entrez/query.fcgi?db=gene&cmd=Retrieve&dopt=Graphics&list_uids=16323) | 1.38 |
|  | Mm00433562_m1 | [Gdf1](http://www.ncbi.nlm.nih.gov/entrez/query.fcgi?db=gene&cmd=Retrieve&dopt=Graphics&list_uids=14559) | 0.74 |
|  | Mm03024080_m1 | [Gdf2](http://www.ncbi.nlm.nih.gov/entrez/query.fcgi?db=gene&cmd=Retrieve&dopt=Graphics&list_uids=12165) (Bmp9) | 0.65 |
|  | Mm00433563_m1 | [Gdf3](http://www.ncbi.nlm.nih.gov/entrez/query.fcgi?db=gene&cmd=Retrieve&dopt=Graphics&list_uids=14562) (Vgr-2) | 2.07 |
|  | Mm00433564_m1 | [Gdf5](http://www.ncbi.nlm.nih.gov/entrez/query.fcgi?db=gene&cmd=Retrieve&dopt=Graphics&list_uids=14563)(CDMP-1) | 1.68 |
|  | Mm03024074_m1 | [Gdf6](http://www.ncbi.nlm.nih.gov/entrez/query.fcgi?db=gene&cmd=Retrieve&dopt=Graphics&list_uids=242316) | 0.78 |
|  | Mm00807130_m1 | [Gdf7](http://www.ncbi.nlm.nih.gov/entrez/query.fcgi?db=gene&cmd=Retrieve&dopt=Graphics&list_uids=238057) | 0.9 |
|  | Mm00443040_m1 | [Nodal](http://www.ncbi.nlm.nih.gov/entrez/query.fcgi?db=gene&cmd=Retrieve&dopt=Graphics&list_uids=18119) | 1.85 |
|  | Mm01178819_m1 | [Tgfb1](http://www.ncbi.nlm.nih.gov/entrez/query.fcgi?db=gene&cmd=Retrieve&dopt=Graphics&list_uids=21803) | 1.24 |
|  | Mm00436955_m1 | [Tgfb2](http://www.ncbi.nlm.nih.gov/entrez/query.fcgi?db=gene&cmd=Retrieve&dopt=Graphics&list_uids=21808) | 1.37 |
|  | Mm00436960_m1 | [Tgfb3](http://www.ncbi.nlm.nih.gov/entrez/query.fcgi?db=gene&cmd=Retrieve&dopt=Graphics&list_uids=21809) | 0.73 |
|  | Mm00514535_m1 | [Dcn](http://www.ncbi.nlm.nih.gov/entrez/query.fcgi?db=gene&cmd=Retrieve&dopt=Graphics&list_uids=13179) | 0.95 |
|  | Mm00438615_m1 | [Lefty1](http://www.ncbi.nlm.nih.gov/entrez/query.fcgi?db=gene&cmd=Retrieve&dopt=Graphics&list_uids=13590) | 1.87 |
|  | Mm01226406_m1 | [Ltbp1](http://www.ncbi.nlm.nih.gov/entrez/query.fcgi?db=gene&cmd=Retrieve&dopt=Graphics&list_uids=268977) | 1.44 |
|  | Mm01307370_m1 | [Ltbp2](http://www.ncbi.nlm.nih.gov/entrez/query.fcgi?db=gene&cmd=Retrieve&dopt=Graphics&list_uids=16997) | 2.75 |
|  | Mm01173079_m1 | [Ltbp4](http://www.ncbi.nlm.nih.gov/entrez/query.fcgi?db=gene&cmd=Retrieve&dopt=Graphics&list_uids=108075) | 1.2 |
|  | Mm00476456_s1 | [Nog](http://www.ncbi.nlm.nih.gov/entrez/query.fcgi?db=gene&cmd=Retrieve&dopt=Graphics&list_uids=18121) | 0.87 |
|  | Mm00493648_m1 | [Tgfbi](http://www.ncbi.nlm.nih.gov/entrez/query.fcgi?db=gene&cmd=Retrieve&dopt=Graphics&list_uids=21810) | 1.16 |
|  | Mm01254088_m1 | [Tgfbrap1](http://www.ncbi.nlm.nih.gov/entrez/query.fcgi?db=gene&cmd=Retrieve&dopt=Graphics&list_uids=73122) | 0.77 |
|  | Mm01335418_m1 | [Thbs1](http://www.ncbi.nlm.nih.gov/entrez/query.fcgi?db=gene&cmd=Retrieve&dopt=Graphics&list_uids=21825)(TSP-1) | 0.46 |
| TGFβ Superfamily Receptors | Mm01331069_m1 | [Acvr1](http://www.ncbi.nlm.nih.gov/entrez/query.fcgi?db=gene&cmd=Retrieve&dopt=Graphics&list_uids=11477) (ALK2) | 2.14 |
|  | Mm00431657_m1 | [Acvr2a](http://www.ncbi.nlm.nih.gov/entrez/query.fcgi?db=gene&cmd=Retrieve&dopt=Graphics&list_uids=11480) | 1.66 |
|  | Mm00437432_m1 | [Acvrl1](http://www.ncbi.nlm.nih.gov/entrez/query.fcgi?db=gene&cmd=Retrieve&dopt=Graphics&list_uids=11482) (Alk1) | 0.85 |
|  | Mm00477650_m1 | [Bmpr1a](http://www.ncbi.nlm.nih.gov/entrez/query.fcgi?db=gene&cmd=Retrieve&dopt=Graphics&list_uids=12166) (ALK3) | 1.32 |
|  | Mm01312643_m1 | [Bmpr1b](http://www.ncbi.nlm.nih.gov/entrez/query.fcgi?db=gene&cmd=Retrieve&dopt=Graphics&list_uids=12167) (Alk6) | 0.93 |
|  | Mm00432134_m1 | [Bmpr2](http://www.ncbi.nlm.nih.gov/entrez/query.fcgi?db=gene&cmd=Retrieve&dopt=Graphics&list_uids=12168) | 0.98 |
|  | Mm00436964_m1 | [Tgfbr1](http://www.ncbi.nlm.nih.gov/entrez/query.fcgi?db=gene&cmd=Retrieve&dopt=Graphics&list_uids=21812) (ALK5) | 1.35 |
|  | Mm00436977_m1 | [Tgfbr2](http://www.ncbi.nlm.nih.gov/entrez/query.fcgi?db=gene&cmd=Retrieve&dopt=Graphics&list_uids=21813) | 1.72 |
|  | Mm00803538_m1 | [Tgfbr3](http://www.ncbi.nlm.nih.gov/entrez/query.fcgi?db=gene&cmd=Retrieve&dopt=Graphics&list_uids=21814) | 0.83 |
| Transcription Factors & Regulators | Mm00438426_g1 | [Dlx2](http://www.ncbi.nlm.nih.gov/entrez/query.fcgi?db=gene&cmd=Retrieve&dopt=Graphics&list_uids=13392) | 1.61 |
|  | Mm00487425_m1 | [Fos](http://www.ncbi.nlm.nih.gov/entrez/query.fcgi?db=gene&cmd=Retrieve&dopt=Graphics&list_uids=14281) | 0.58 |
|  | Mm00495062_s1 | [Jun](http://www.ncbi.nlm.nih.gov/entrez/query.fcgi?db=gene&cmd=Retrieve&dopt=Graphics&list_uids=16476) | 1.42 |
|  | Mm04243546_s1 | [Junb](http://www.ncbi.nlm.nih.gov/entrez/query.fcgi?db=gene&cmd=Retrieve&dopt=Graphics&list_uids=16477) | 1.26 |
|  | Mm00484721_m1 | [Smad1](http://www.ncbi.nlm.nih.gov/entrez/query.fcgi?db=gene&cmd=Retrieve&dopt=Graphics&list_uids=17125) (Madh1) | 0.85 |
|  | Mm00487530_m1 | [Smad2](http://www.ncbi.nlm.nih.gov/entrez/query.fcgi?db=gene&cmd=Retrieve&dopt=Graphics&list_uids=17126) (Madh2) | 0.79 |
|  | Mm01170760_m1 | [Smad3](http://www.ncbi.nlm.nih.gov/entrez/query.fcgi?db=gene&cmd=Retrieve&dopt=Graphics&list_uids=17127) (Madh3) | 1.67 |
|  | Mm03023996_m1 | [Smad4](http://www.ncbi.nlm.nih.gov/entrez/query.fcgi?db=gene&cmd=Retrieve&dopt=Graphics&list_uids=17128) (Madh4) | **0.22** |
|  | Mm03024001_g1 | [Smad5](http://www.ncbi.nlm.nih.gov/entrez/query.fcgi?db=gene&cmd=Retrieve&dopt=Graphics&list_uids=17129) (Madh5) | 1.78 |
|  | Mm00484741_m1 | [Smad7](http://www.ncbi.nlm.nih.gov/entrez/query.fcgi?db=gene&cmd=Retrieve&dopt=Graphics&list_uids=17131) | **4.78** |
|  | Mm00486317_s1 | [Sox4](http://www.ncbi.nlm.nih.gov/entrez/query.fcgi?db=gene&cmd=Retrieve&dopt=Graphics&list_uids=20677) | 1.48 |
|  | Mm00439518_m1 | [Stat1](http://www.ncbi.nlm.nih.gov/entrez/query.fcgi?db=gene&cmd=Retrieve&dopt=Graphics&list_uids=20846) | 0.71 |
|  | Mm01275580_m1 | [Tgfb1i1](http://www.ncbi.nlm.nih.gov/entrez/query.fcgi?db=gene&cmd=Retrieve&dopt=Graphics&list_uids=21804) | 0.62 |
| SMAD Target Genes | Mm00515324_m1 | [Atf4](http://www.ncbi.nlm.nih.gov/entrez/query.fcgi?db=gene&cmd=Retrieve&dopt=Graphics&list_uids=11911) | 2.8 |
|  | Mm00503759_m1 | [Cdkn1a](http://www.ncbi.nlm.nih.gov/entrez/query.fcgi?db=gene&cmd=Retrieve&dopt=Graphics&list_uids=12575) (p21Cip1,Waf1) | 0.57 |
|  | Mm00438168_m1 | [Cdkn1b](http://www.ncbi.nlm.nih.gov/entrez/query.fcgi?db=gene&cmd=Retrieve&dopt=Graphics&list_uids=12576) (p27Kip1) | 0.94 |
|  | Mm00483241_m1 | [Cdkn2b](http://www.ncbi.nlm.nih.gov/entrez/query.fcgi?db=gene&cmd=Retrieve&dopt=Graphics&list_uids=12579) (p15INK4b) | 0.6 |
|  | Mm00515678_m1 | [Emp1](http://www.ncbi.nlm.nih.gov/entrez/query.fcgi?db=gene&cmd=Retrieve&dopt=Graphics&list_uids=13730) | 0.54 |
|  | Mm00435121_g1 | [Gadd45b](http://www.ncbi.nlm.nih.gov/entrez/query.fcgi?db=gene&cmd=Retrieve&dopt=Graphics&list_uids=17873) | 0.88 |
|  | Mm00650681_g1 | [Gsc](http://www.ncbi.nlm.nih.gov/entrez/query.fcgi?db=gene&cmd=Retrieve&dopt=Graphics&list_uids=14836) | 0.73 |
|  | Mm00445604_m1 | [Herpud1](http://www.ncbi.nlm.nih.gov/entrez/query.fcgi?db=gene&cmd=Retrieve&dopt=Graphics&list_uids=64209) | 0.89 |
|  | Mm00516395_m1 | [Ifrd1](http://www.ncbi.nlm.nih.gov/entrez/query.fcgi?db=gene&cmd=Retrieve&dopt=Graphics&list_uids=15982) | 0.76 |
|  | Mm00439561_m1 | [Igf1](http://www.ncbi.nlm.nih.gov/entrez/query.fcgi?db=gene&cmd=Retrieve&dopt=Graphics&list_uids=16000) | 1.69 |
|  | Mm00515156_m1 | [Igfbp3](http://www.ncbi.nlm.nih.gov/entrez/query.fcgi?db=gene&cmd=Retrieve&dopt=Graphics&list_uids=16009) | 0.4 |
|  | Mm01210733_m1 | [Il6](http://www.ncbi.nlm.nih.gov/entrez/query.fcgi?db=gene&cmd=Retrieve&dopt=Graphics&list_uids=16193) | 0.78 |
|  | Mm00440677_m1 | [Pdgfb](http://www.ncbi.nlm.nih.gov/entrez/query.fcgi?db=gene&cmd=Retrieve&dopt=Graphics&list_uids=18591) | 0.48 |
|  | Mm01204469_m1 | [Serpine1](http://www.ncbi.nlm.nih.gov/entrez/query.fcgi?db=gene&cmd=Retrieve&dopt=Graphics&list_uids=18787) (PAI-1) | 0.76 |
|  | Mm01283606_m1 | [Tnfsf10](http://www.ncbi.nlm.nih.gov/entrez/query.fcgi?db=gene&cmd=Retrieve&dopt=Graphics&list_uids=22035) (Trail) | 0.55 |
|  | Mm00493632_g1 | [Tsc22d1](http://www.ncbi.nlm.nih.gov/entrez/query.fcgi?db=gene&cmd=Retrieve&dopt=Graphics&list_uids=21807) (Tgfb1i4) | 0.80 |
|  | Mm00775963_g1 | [Id1](http://www.ncbi.nlm.nih.gov/entrez/query.fcgi?db=gene&cmd=Retrieve&dopt=Graphics&list_uids=15901) | 0.51 |
|  | Mm00711781_m1 | [Id2](http://www.ncbi.nlm.nih.gov/entrez/query.fcgi?db=gene&cmd=Retrieve&dopt=Graphics&list_uids=15902) | 0.63 |
|  | Mm03053974_s1 | [Bambi](http://www.ncbi.nlm.nih.gov/entrez/query.fcgi?db=gene&cmd=Retrieve&dopt=Graphics&list_uids=68010) | 1.03 |
|  | Mm00459279_m1 | [Bmper](http://www.ncbi.nlm.nih.gov/entrez/query.fcgi?db=gene&cmd=Retrieve&dopt=Graphics&list_uids=73230) | 0.38 |
|  | Mm00514820_m1 | [Mecom](http://www.ncbi.nlm.nih.gov/entrez/query.fcgi?db=gene&cmd=Retrieve&dopt=Graphics&list_uids=14013) | 1.24 |
|  | Mm00514982_m1 | [Fst](http://www.ncbi.nlm.nih.gov/entrez/query.fcgi?db=gene&cmd=Retrieve&dopt=Graphics&list_uids=14313) | 0.54 |
|  | Mm00447054_m1 | [Plau](http://www.ncbi.nlm.nih.gov/entrez/query.fcgi?db=gene&cmd=Retrieve&dopt=Graphics&list_uids=18792) (uPA) | 0.42 |
|  | Mm01213405_m1 | [Runx1](http://www.ncbi.nlm.nih.gov/entrez/query.fcgi?db=gene&cmd=Retrieve&dopt=Graphics&list_uids=12394) (AML1) | 0.69 |
|  | Mm01205111_m1 | Smurf1 | 0.67 |
| Extracellular Matrix (ECM) & Cell Adhesion Molecules | Mm03413826_mH | [Bglap2](http://www.ncbi.nlm.nih.gov/entrez/query.fcgi?db=gene&cmd=Retrieve&dopt=Graphics&list_uids=12097) | 0.57 |
|  | Mm00468256_m1 | [Eng](http://www.ncbi.nlm.nih.gov/entrez/query.fcgi?db=gene&cmd=Retrieve&dopt=Graphics&list_uids=13805) (Evi-1) | 0.70 |
| Cellular & Developmental Processes | Mm01605857_g1 | [Tdgf1](http://www.ncbi.nlm.nih.gov/entrez/query.fcgi?db=gene&cmd=Retrieve&dopt=Graphics&list_uids=21667) | 1.4 |
|  | Mm01172799_g1 | [Amh](http://www.ncbi.nlm.nih.gov/entrez/query.fcgi?db=gene&cmd=Retrieve&dopt=Graphics&list_uids=11705) | 0.33 |
|  | Mm01300271_g1 | [Amhr2](http://www.ncbi.nlm.nih.gov/entrez/query.fcgi?db=gene&cmd=Retrieve&dopt=Graphics&list_uids=110542) | 0.49 |
|  | Mm01315636_m1 | [Bmp1](http://www.ncbi.nlm.nih.gov/entrez/query.fcgi?db=gene&cmd=Retrieve&dopt=Graphics&list_uids=12153) | 0.75 |
|  | Mm01340178_m1 | [Bmp2](http://www.ncbi.nlm.nih.gov/entrez/query.fcgi?db=gene&cmd=Retrieve&dopt=Graphics&list_uids=12156) | 0.42 |
|  | Mm03024297_s1 | [Bmp3](http://www.ncbi.nlm.nih.gov/entrez/query.fcgi?db=gene&cmd=Retrieve&dopt=Graphics&list_uids=110075) | 0.62 |
|  | Mm00432087_m1 | [Bmp4](http://www.ncbi.nlm.nih.gov/entrez/query.fcgi?db=gene&cmd=Retrieve&dopt=Graphics&list_uids=12159) | 0.73 |
|  | Mm00432089_m1 | [Bmp5](http://www.ncbi.nlm.nih.gov/entrez/query.fcgi?db=gene&cmd=Retrieve&dopt=Graphics&list_uids=12160) | 0.61 |
|  | Mm01332882_m1 | [Bmp6](http://www.ncbi.nlm.nih.gov/entrez/query.fcgi?db=gene&cmd=Retrieve&dopt=Graphics&list_uids=12161) | 0.96 |
|  | Mm00432102_m1 | [Bmp7](http://www.ncbi.nlm.nih.gov/entrez/query.fcgi?db=gene&cmd=Retrieve&dopt=Graphics&list_uids=12162) | 0.59 |
|  | Mm01170557_g1 | [Chrd](http://www.ncbi.nlm.nih.gov/entrez/query.fcgi?db=gene&cmd=Retrieve&dopt=Graphics&list_uids=12667) | 1.85 |
|  | Mm01302043_g1 | [Col1a1](http://www.ncbi.nlm.nih.gov/entrez/query.fcgi?db=gene&cmd=Retrieve&dopt=Graphics&list_uids=12842) | 1.36 |
|  | Mm01165181_m1 | [Col1a2](http://www.ncbi.nlm.nih.gov/entrez/query.fcgi?db=gene&cmd=Retrieve&dopt=Graphics&list_uids=12843) | 0.64 |
